# Comparing methods for controlled capture and quantification of pollen in *Cannabis sativa*

**DOI:** 10.1002/aps3.11389

**Published:** 2020-09-30

**Authors:** Sydney B. Wizenberg, Arthur E. Weis, Lesley G. Campbell

**Affiliations:** ^1^ Department of Chemistry and Biology Ryerson University Toronto Ontario Canada; ^2^ Department of Ecology and Evolutionary Biology University of Toronto Toronto Ontario Canada

**Keywords:** *Cannabis sativa*, controlled crosses, hemp, light spectroscopy, pollen containment, wind pollination

## Abstract

**Premise:**

Precise pollen collection methods are necessary for crop breeding, but anemophilous pollen is notoriously difficult to capture and control. Here we compared a variety of methods for the controlled capture of cannabis pollen, intended to ease the process of cross‐fertilization for breeding this wind‐pollinated plant, and measured the utility of light spectroscopy for quantifying relative pollen yield.

**Methods and Results:**

In two independent trials, we compared a control method of pollen collection (hand collection) to either vacuum‐, water‐, or bag‐collection methods. We used visible light spectroscopy to quantify relative pollen yield, and validated this approach using microscopic pollen counts. We determined that pollen yield was highest when using hand collection or vacuum collection, but efficiency did not differ significantly among methods.

**Conclusions:**

To maximize yield, pollen should be collected by hand or vacuum, but all collection methods were equally efficient in a relative sense because yield increased with collection time. We also found that light spectroscopy is an accurate and rapid method of quantifying pollen abundance (*R* ^2^ = 0.86) in a liquid suspension.

*Cannabis sativa* L. (Cannabaceae) is a dioecious plant, producing male and female flowers on separate unisexual individuals (Sinoto, 1929; Valle et al., 1968). Although both male and female plants are capable of producing cannabinoids in equal concentrations (Valle et al., 1968), female plants produce greater floral biomass than male plants (Ohlsson et al., 1971) and thus are exclusively used in commercial marijuana production facilities. Moreover, after pollination, female plants alter their relative investment in phytochemicals by reducing the production of secondary metabolites like cannabinoids, flavonoids, and terpenoids (Pijlman et al., 2005). In the absence of pollen, stigmas on female plants continue to grow and thus produce more surface area on which cannabinoids can be produced (Small and Naraine, 2016). Because of this negative impact of pollination on cannabinoid yield, industrial growers rarely maintain male plants in production facilities, and instead propagate their stock of female plants by vegetative cloning (Flores‐Sanchez and Verpoorte, 2008; Decorte, 2010). However, the “mother” plants used to produce clones eventually become non‐regenerative and new mother plants are grown from seed, which necessitates pollination (Valle et al., 1968). Therefore, careful consideration must be given as to the most effective and efficient ways to collect pollen for controlled crosses while preventing pollen escape into production areas.

Cannabis is anemophilous (wind‐pollinated) (Small and Antle, 2003), and therefore relies on air movement for pollen transfer from male to female plants, sometimes across long distances (Small and Antle, 2003). Pollen dispersal mechanisms often reflect pollen ornamentation, as seen in *C. sativa*’s smooth exine layer, triporiate (three aperture) morphology, and low mass—features intended to maximize pollen dispersal distance and chance of successful ovule fertilization (Hesse et al., 2009). The aerodynamic morphology of *C. sativa*’s pollen highlights the difficulty associated with controlling its movement, as any airflow following anther dehiscence can result in pollen movement, a frequent issue when studying dispersal in anemophilous species (Whitehead, 1969, 1983). It is therefore important to determine the most efficient method of capturing wind‐borne pollen upon anthesis, in terms of both the number of pollen grains collected and the time spent collecting pollen.

Procedures for controlled pollen capture are typically required in crop breeding programs to ensure precise knowledge of paternity so as to breed progeny with preferred traits (Richey, 1950; Briggs and Knowles, 1967; Allard, 1999). For example, standard methods for maize breeding were established in the early 1900s, with an abundance of literature outlining the procedure for controlled crosses (Hopkins et al., 1905; Borgeson, 1943; Jones and Mangelsdorf, 1951; Mangelsdorf, 1951). However, because corn is monecious, breeding procedures prioritize avoidance of self‐fertilization (through de‐tasseling or tassel bagging), with controlled capture of pollen samples as a secondary goal (Borgeson, 1943; Mangelsdorf, 1951). Studies on controlled pollen capture in other species have developed methods based on species‐specific traits, such as the clipping of large anthers in *Eucalyptus* L’Hér. (Griffin et al., 1982). Although some literature related to maximizing pollen capture from trees describes methods that may be applied to cannabis (Copes et al., 1991), these would require modification based on the scale of collection and organismal size. In addition, most research on determining optimal methods for controlled pollination relates to pollen storage and germination conditions (van der Maas et al., 1993; Daher et al., 2009; Alcaraz et al., 2011; Conner, 2011) rather than optimizing controlled pollen capture.

One of the largest barriers to comparing the efficiency of pollen collection methods is quantifying relative pollen yield. Previous research on pollen production in cannabis, which estimated the number of pollen grains per anther, relied on hemocytometers (Rana and Choudhary, 2010), a method frequently employed for counting pollen grains (Godini, 1981). More broadly, light scattering as a method for rapidly estimating particle abundance is well documented (Debye, 1944; Mullaney et al., 1969; Cross et al., 2007; Kawashima et al., 2007), and laser scattering has been used to analyze the physical properties of pollen grains (Matsuda and Kawashima, 2018). Relative to direct pollen counting using a hemocytometer, visible light spectroscopy could allow for the rapid quantification of particles in a liquid suspension.

Here, we compared several existing methods used to collect pollen in other species, i.e., hand collection (Abraham and Nair, 1990; Chautá‐Mellizo et al., 2012), vacuum collection (Copes et al., 1991; Daher et al., 2009), bag collection (Owens et al., 1981; McAdam et al., 1987; Takaso and Owens, 1995), and water collection (Griffin et al., 1982; Hopping and Hacking, 1982), and explored their use in cannabis. Notably, we could not find any peer‐reviewed publications that directly compared the efficiency of such methods (especially in wind‐dispersed species), although many have examined pollen collection using a single methodology (MacDaniels, 1930; Baldet and Philippe, 1993; Holcroft and Allan, 1994; Gan‐Mor et al., 1995, 1999; Vaknin et al., 2001). Collecting pollen in large quantities may be of use in commercial crop breeding programs, especially when creating a repository of genetic stock for later use, and as such, we were interested in both the relative yield and efficiency of various methods. Hand collection, while simple in practice, may be inefficient because, in cannabis, it relies on pollen removal from individual flowers, one by one. Comparatively, vacuum collection may be more efficient (in terms of grains collected per unit of time) but could be prone to sample contamination if male plants are not properly isolated from each other. Bag collection, similar to vacuum collection, is efficient, but the plant must be able to hold up bags; in the case of cannabis, male plants are so diminutive, and the flowers are so dispersed on a plant, that this is a difficult endeavor (Small et al., 2003). Bag collection also could result in reduced yield if issues such as static charge of pollen grains are not sufficiently addressed (Durham, 1946; Schroeder, 1995). To assess the relative efficiency (and practicality) of each pollen collection method and develop an optimal procedure for use in cannabis, we asked the following:

1. Can visible light spectroscopy effectively quantify relative yield when a sample of pollen is suspended in water compared to traditional approaches?
2. Which pollen collection method provides the highest yield?
3. Which pollen collection method is most efficient in terms of yield per unit of time?

## METHODS AND RESULTS

### Plant genotypes and growth conditions

We used two hemp cultivars of *C. sativa* (CFX‐1 and CFX‐2), both possessing an expected total tetrahydrocannabinol (THC) content of less than 0.01%; we grew CFX‐1 in the first trial, and CFX‐2 in the second trial (Hemp Genetics International, Saskatoon, Saskatchewan, Canada). Following germination in a two‐tier terracotta germination pot (ANVÄNDBAR Sprouter; IKEA, Delft, The Netherlands), which took three days, we planted the seedlings in SC‐10 containers (Stuewe and Sons Inc., Tangent, Oregon, USA) filled with 200 mL of PRO‐MIX BX mycorrhizae peat moss growing medium (Premier Tech, Rivière‐du‐Loup, Quebec, Canada). A week later, we transplanted seedlings into 1‐L pots (AgricUltra, Stoney Creek, Ontario, Canada) filled with the same growing medium. We applied 250 mL of filtered water twice weekly and applied 250 mL of 0.4% diluted Miracle‐Gro (10‐10‐10 NPK; ScottsMiracle‐Gro, Marysville, Ohio, USA) once weekly. For four weeks, plants grew under 24‐h lighting from high‐pressure sodium fixtures (Gavita 1000W; Gavita North America, Vancouver, Washington, USA). Male floral development was visible in the third week, and we selected early‐flowering males for use in our experiments to minimize variability in the number of inflorescences (and subsequent pollen produced) on each plant. We pruned the apical meristems of male plants twice, once in week 3 and once in week 4, to promote increased branching and thus inflorescence growth. After approximately four weeks, we switched plants to 12‐h lighting to induce anthesis under visible spectrum LED fixtures (HyperRail, 250 W, part #MR18‐4ALBL‐ND‐120V; AgricUltra).

### Experimental methods

**Figure 1:**
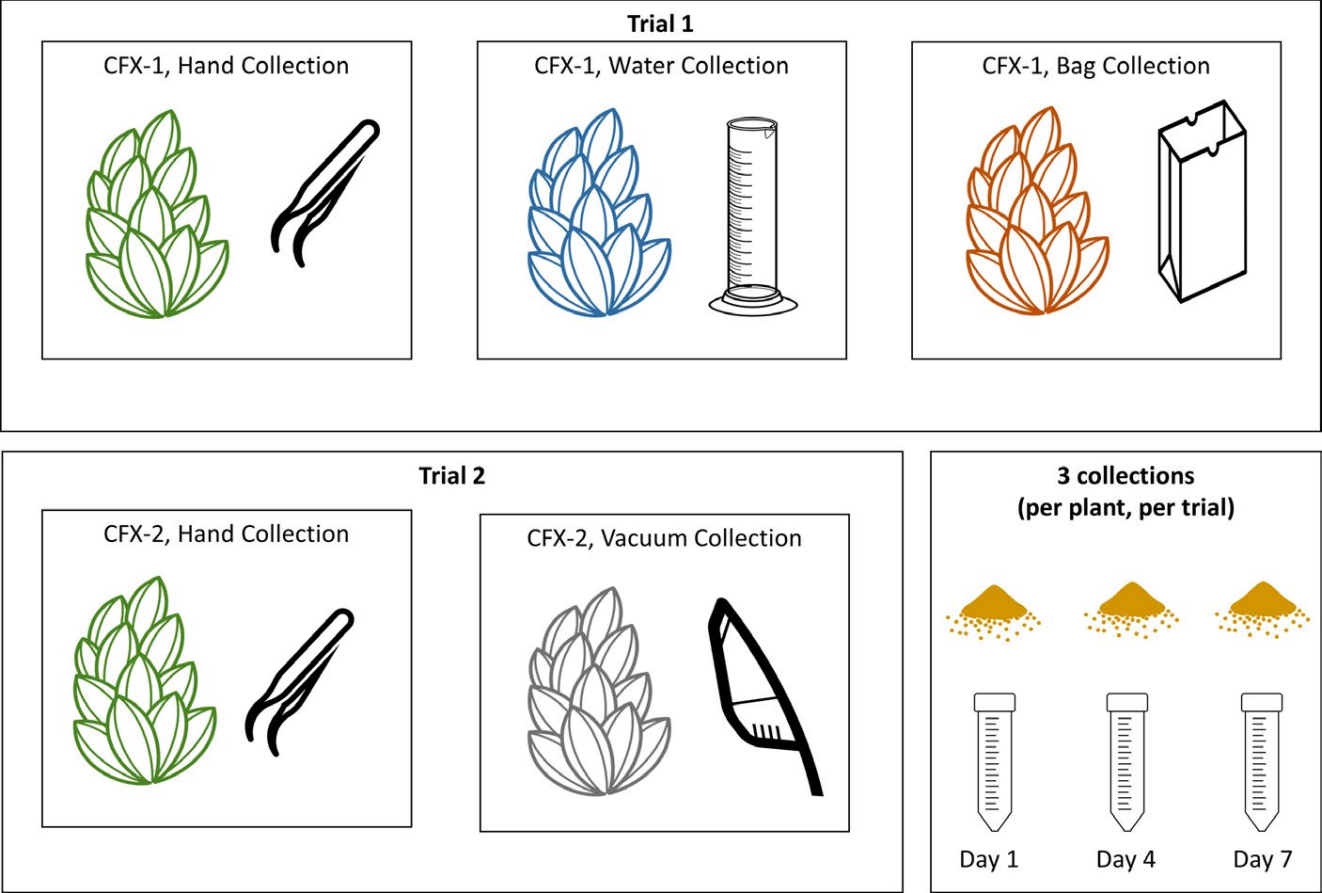
Experimental design across two trials, using two hemp genotypes (CFX‐1, CFX‐2). Icons were openly shared on The Noun Project (https://thenounproject.com) by the following artists: inflorescences, Olena Panasovska; graduated cylinder, Victoria Codes; paper bag, Ryan Spiering; tweezers, Phuong Hung; vacuum, Daniel Luft; pollen, Michael G. Brown.

In the first trial, we used three pollen collection methods (also referred to as collection treatments): hand collection (control), bag collection, and water collection (Fig. 1). In the second trial, we maintained hand collection as a control and tested vacuum collection. To compare the yield and efficiency of collection methods, we imposed each pollen collection treatment on a randomly selected subset of experimental plants, each of which we collected three times during the course of the trial. Cannabis anthers dehisce non‐concurrently, and as such we initiated pollen collection when at least 33% of visible male flowers were releasing pollen (this date was termed day zero) to ensure there was enough pollen to collect in the context of a breeding program. From each plant, we performed three collections over a seven‐day period, where initial sufficient anther dehiscence occurred on day zero, the first collection occurred on day 1, and subsequent collections on days 4 and 7. In our first trial, we attempted to perform a fourth collection on day 10 but found that by this point the plants were no longer producing enough pollen to warrant a fourth collection from that point onward. All pollen samples were stored at −20°C after collection.

Our collection protocols were as follows, during hand collection, we placed a 50‐mL centrifuge vial (Falcon 50‐mL centrifuge tubes; Thermo Fisher Scientific, Waltham, Massachusetts, USA) at the base of inflorescences with dehiscent anthers and used tweezers to tap or brush the dehiscent flowers, allowing pollen to fall into the collection vial. During the water collection method, we flipped plants upside down and dipped them into a 100‐mL graduated cylinder filled with 50 mL of distilled water to wash all the pollen off the plant. The water sample containing pollen was then transferred into a 50‐mL centrifuge vial for storage. During bag collection, we placed brown paper bags (41 cm × 11 cm × 5 cm) on the plants and loosely tied the base of the bag using twine. We then flipped the plants upside down and lightly shook them for 10 s to encourage dehiscence of pollen, after which we untied the twine from the base of the bag to remove the plant, and quickly sealed the bag to prevent pollen loss.

After the first trial was completed and it appeared that bag and water collection methods would likely be less successful and/or efficient than hand collection, we focused on comparing the efficiency of vacuum to hand collection in the second trial. We retrofitted small paper cups (9 oz, DR‐1000765, Dixie; Koch Industries, Wichita, Kansas, USA) to act as filters inside a hand‐held vacuum (4‐W mini portable vacuum; Honk Electronic Co. Ltd., Shenzhen, China) by reducing the height of the cup to 2 cm and poking four holes (using a hole punch) along the cup’s sides (0.63‐cm diameter) to allow a small amount of airflow through the filter. Retrofitted cup filters were then placed inside the body of the vacuum, between the nozzle and the motor, intercepting and storing all of the vacuumed particles. Pollen was then collected by vacuuming the leaves and inflorescences of male plants, after which the filter was carefully removed and transferred to plastic containers for storage.

### Measuring yield

Using a stopwatch, we recorded the time spent collecting pollen (in seconds) for each method. For the treatments that did not involve water collection, we mixed each pollen sample into 50 mL of distilled water, and then vortexed (Corning LSE vortex mixer, 230 V, product #6776; Corning Inc., Corning, New York, USA) the samples for 30 s to create a liquid suspension with a consistent distribution of pollen grains. To quantify the relative grain density in each sample, we used visible light spectroscopy, employing the absorption reading as a response variable. We pipetted 2 mL of the vortexed suspension into a 3‐mL cuvette and then used the light spectrometer (Pasco wireless spectrometer and fluorometer, PS‐2600; Pasco Scientific, Roseville, California, USA) to quantify the proportion of light that was reflected by the sample. We ultimately chose 425 nm as the reflectance wavelength for the absorption reading by testing multiple wavelengths on the first sample and then identifying the wavelength region that corresponded to the peak in the absorption curve. To verify that our light spectroscopy readings were truly indicative of the amount of pollen in each sample, we pipetted 5 μL of the suspension in each cuvette onto a glass slide and used a light microscope at 10× magnification to count the number of grains contained in the sample. Counting was done using a hand‐held tally counter (Uline hand‐held tally counter; Uline, Pleasant Prairie, Wisconsin, USA), and counting extended across the entire length and width of the slide cover (22 mm × 22 mm). To determine if any of the pollen grains had burst and, if so, what proportion of the total sample they represented, we counted the number of burst grains on each slide using a second hand‐held tally counter. All data used in this paper are provided Appendix S1.

### Data analysis

All analyses were run in R version 3.6.0 (using the *stats* package 2019‐04‐06; R Core Team, 2019). To test if spectroscopy readings were correlated with microscopy‐derived pollen count data, we used a linear regression model. We used the adjusted *R* ^2^ value and correlation of the linear model to evaluate how well reflectance predicts pollen counts. A strong positive relationship was confirmed (see below), and so we used this method to compare yield and efficiency for the four different collection protocols.

We compared the effectiveness of the collection methods using repeated measures analysis of variance (ANOVA), where method was a fixed effect, collection event (day 1, 4, and 7) was the repeated measure, and plant ID treatment was used as an error term. We log‐transformed the response variables, i.e., the spectroscopy reading (yield) and efficiency (yield divided by time spent collecting), to satisfy the assumption of normally distributed residuals. We then compared transformed estimates of pollen yield and collection efficiency with a repeated‐measures multivariate ANOVA (MANOVA) (analyzing both yield and efficiency as the response variables) followed by individual repeated‐measures ANOVAs (analyzing yield and efficiency as response variables independently) when factors were found to be statistically significant, using the manova() and aov() functions. Any experimental factors that were determined to be statistically significant underwent subsequent post‐hoc analysis using Tukey’s honest significant difference test (using the TukeyHSD() function in the R *stats* package, version 3.6.0).

### Results

**Figure 2:**
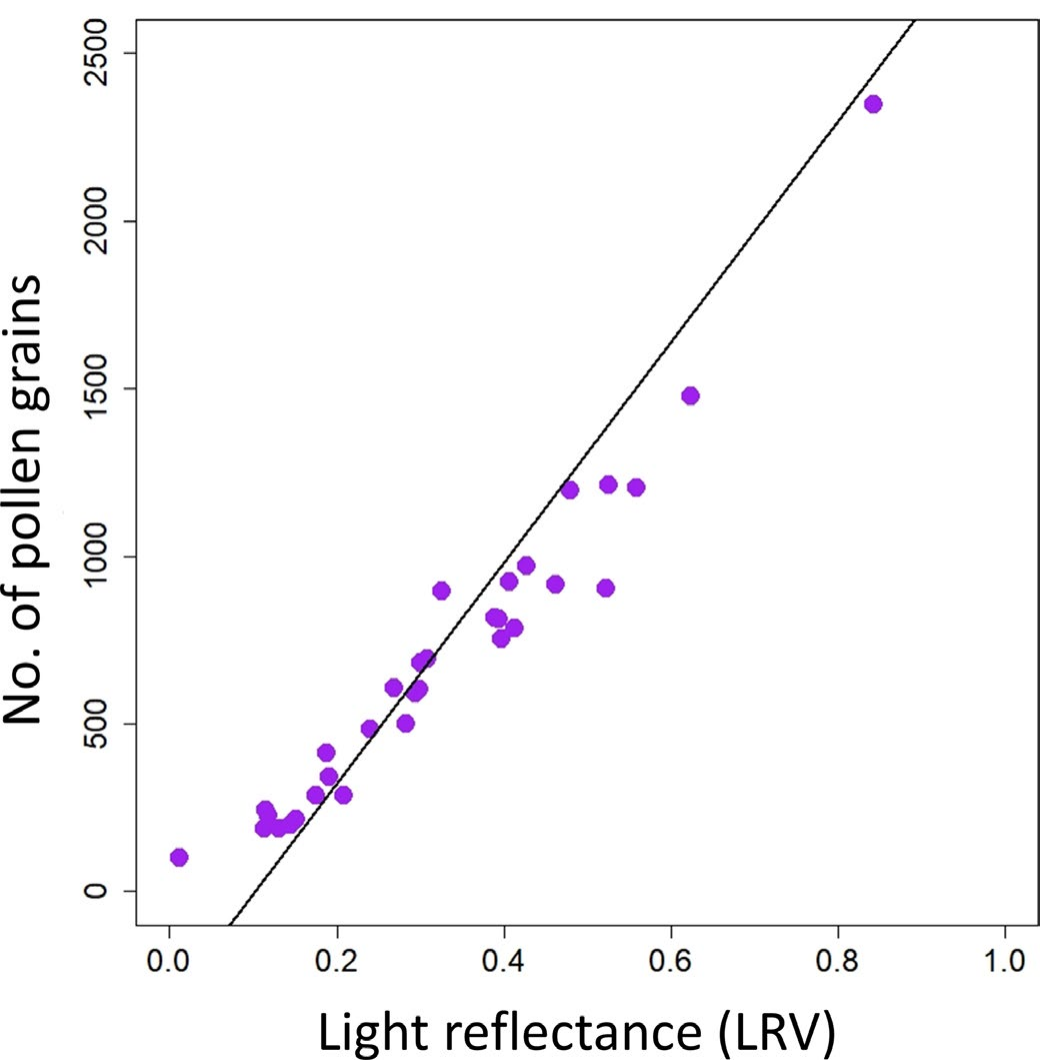
The linear relationship between the number of pollen grains counted in a 5‐μL liquid suspension using microscopy and the reflectance of the same sample using visible light spectroscopy at a wavelength of 425 nm. Note: the visible light spectrometer was standardized to 3; LRV is the proportion of light reflected by the suspension.

Visible light spectroscopy readings strongly predicted microscopy‐derived pollen counts in liquid samples (adjusted *R* ^2^ = 0.86, df = 31, *F* = 190.4, *P* < 0.001; Fig. 2), indicating that this method accurately quantifies pollen abundance. Correlation between the two variables was high (*r* = 0.93), implying that there was a strong linear relationship between pollen counts and light spectroscopy readings. On average, only 0.88% (±0.56%) of pollen grains had burst.

**Figure 3:**
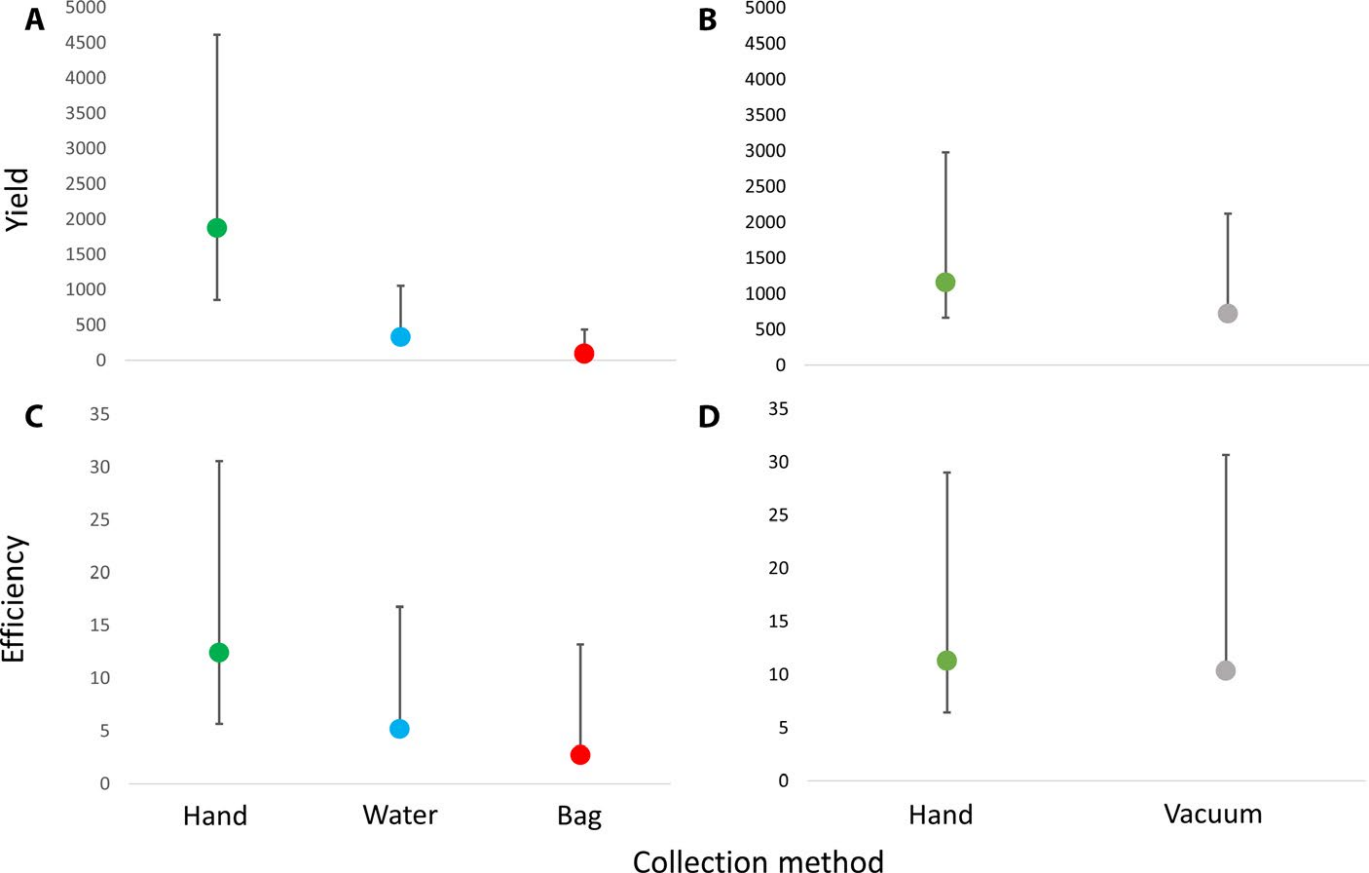
Plots of mean values (±SD) of back‐transformed reflectance data converted to pollen concentration values for an equivalent sample (5 μL). Yield is the average number of pollen grains contained in a sample; efficiency is the same value divided by the average time spent collecting for each method. (A) Yield in trial 1 (hand, water, and bag collection); (B) yield in trial 2 (hand and vacuum collection); (C) efficiency in trial 1; (D) efficiency in trial 2. In (A), hand collection differed significantly from both water and bag collection.

Both trial 1 and trial 2 showed significant differences between collection protocols in the initial repeated measures MANOVAs (Table 1). Trial 1, which compared hand, bag, and water collection, showed significant differences between methods for relative yield (Fig. 3A), with post‐hoc analysis revealing that hand collection yielded significantly more pollen than the other two methods (234% increase over water collection [*P* < 0.001], and a 421% increase over bag collection [*P* < 0.001]). Water collection resulted in a somewhat higher yield than bag collection (56% increase, *P* = 0.01; Fig. 3A). Collection yield did not differ across time points, nor was there a time point‐by‐method interaction (Table 1). Collection efficiency (relative yield divided by time spent collecting) was not affected by collection method, time, or their interaction (Fig. 3C, Table 1). Trial 2, which compared hand collection to vacuum collection, showed no significant influence of collection method, time, or their interaction for pollen yield or collection efficiency (Table 1), implying that increases in collection time directly resulted in increases in relative yield (Fig. 3B, D).

**Table 1:** Repeated‐measures analysis of two trials comparing pollen collection methods using two estimates of success—(A) the relative abundance of pollen collected (natural log‐transformed for trial 1) and (B) the efficiency of the pollen collection method, estimated as the relative abundance of pollen collected scaled by collection time (natural log‐transformed for both trials)—and their response to the pollen collection method.^a^

Model^b^	Fixed effects	F (df)	*P* value
Trial 1			
MANOVA	CM	9.40 (4, 38)	**<0.001**
	CE	0.47 (4, 62)	0.76
	CE × CM	1.57 (8, 62)	0.15
ANOVA (abundance)	CM	31.05 (2, 19)	**<0.001**
	CE	0.22 (2, 31)	0.80
	CE × CM	1.47 (4, 31)	0.24
ANOVA (efficiency)	CM	1.02 (2, 19)	0.38
	CE	0.13 (2, 31)	0.88
	CE × CM	1.24 (4, 31)	0.32
Trial 2			
MANOVA	CM	4.95 (2, 15)	**0.02**
	CE	1.10 (4, 60)	0.37
	CE × CM	0.95 (4, 60)	0.44
ANOVA (abundance)	CM	2.65 (1, 16)	0.12
	CE	1.10 (2, 30)	0.36
	CE × CM	0.91 (2, 30)	0.41
ANOVA (efficiency)	CM	0.26 (1, 16)	0.62
	CE	0.22 (2, 30)	0.81
	CE × CM	1.90 (2, 30)	0.17

Note

CE = collection event; CM = collection method.

^a^ Collection event and its interaction with collection method are used as the repeated measure. Significant *P* values are bolded.

^b^ Trial 1: hand collection vs. water collection vs. bag collection; Trial 2: hand collection vs. vacuum collection.

## CONCLUSIONS

Artificial selection for preferential traits in wind‐pollinated species like cannabis critically depends upon effective and efficient methods for pollen collection and storage so as to prevent unintended genetic contamination of selected lines (through unintentional collection of pollen from neighboring plants). Similarly, methods for pollen handling are also essential in cannabis production, where growers have conflicting needs: to maximize yield of the current crop, pollen must be excluded from production plants, but to generate future crops, pollen is essential. A gap in the literature comparing the relative success and efficiency of pollen collection methods highlighted the need to explore the often laborious process of mass collection of pollen for controlled cross‐fertilization. A key step is to determine the best method for the controlled capture of pollen. Here we compared the yield and efficiency of multiple collection methods (hand, bag, water, and vacuum), and also compared two approaches (visible light spectroscopy and microscopy) for quantifying the relative pollen yield of different methods. We found that light spectroscopy was an effective method for quickly and easily quantifying the abundance of pollen when suspended in distilled water. Light spectroscopy is a much faster method for quantifying pollen abundance than microscopy and is successful in predicting the pollen abundance in a collection sample. We anticipated this result, as light spectroscopy has often been used for measuring the abundance of particles in a suspension *(*Debye, 1944; Mullaney et al., 1969; Cross et al., 2007), and variations have previously been used on pollen (Kawashima et al., 2007; Matsuda and Kawashima, 2018).

Hand collection resulted in a higher pollen yield than water or bag collection in our first trial, but the efficiency with which they collected pollen did not differ. In the second trial, hand collection and vacuum collection did not differ in their yield or efficiency, implying that they are equally suitable for pollen capture. Bag and water collection did require significantly less time for capture pollen; however, the substantially lower yield (Fig. 3A) inhibits their application as an effective method of controlled capture. We further note that while our vacuum device did not outperform hand collection, improvement of the design to engineer a better filtration system and tailor suction power to individual growers’ needs could improve yield and efficiency. Ultimately, the results of these experiments serve as an important early step in the establishment of a practical framework for breeding cannabis, as well as other economically valuable wind‐pollinated crops.

## Supporting information


**APPENDIX S1.** Supplemental data for the manuscript. Trial 1 and 2 contain the data collected in each respective experiment, and regression data contains microscopic pollen count data and the related spectrometry reading used to create the regression model (Fig. 2).

## Data Availability

The authors confirm that all data underlying the findings are fully available without restriction. All data are included within the manuscript and Appendix S1.
